# Acute rotavirus infection is associated with the induction of circulating memory CD4^+^ T cell subsets

**DOI:** 10.1038/s41598-023-35681-9

**Published:** 2023-06-02

**Authors:** Chikondi Malamba-Banda, Chimwemwe Mhango, Prisca Benedicto-Matambo, Jonathan J. Mandolo, End Chinyama, Orpha Kumwenda, Kayla G. Barnes, Nigel A. Cunliffe, Miren Iturriza-Gomara, Kondwani C. Jambo, Khuzwayo C. Jere

**Affiliations:** 1 Biological Sciences Departments, Malawi University of Science and Technology, Thyolo, Malawi; 2 Malawi Liverpool Wellcome Research Programme (MLW), Blantyre, Malawi; 3 Department of Medical Laboratory Sciences, Faculty of Biomedical Sciences and Health Profession, Kamuzu University of Health Sciences, Blantyre, Malawi; 4 Institute of Infection, Veterinary and Ecological Sciences, University of Liverpool, Liverpool, UK; 5 National Institute for Health and Care Research, Health Protection Research Unit in Gastrointestinal Infections, University of Liverpool, Liverpool, UK; 6 Department of Clinical Sciences, Liverpool School of Tropical Medicine, Liverpool, UK; 7 Harvard TH Chan School of Public Health, Boston, USA; 8 Broad Institute of MIT and Harvard, Cambridge, USA; 9 University of Glasgow, Glasgow, UK

**Keywords:** Cell biology, Immunology

## Abstract

Strong CD4^+^ T cell-mediated immune protection following rotavirus infection has been observed in animal models, but its relevance in humans remains unclear. Here, we characterized acute and convalescent CD4^+^ T cell responses in children who were hospitalized with rotavirus-positive and rotavirus-negative diarrhoea in Blantyre, Malawi. Children presenting with laboratory-confirmed rotavirus infection had higher proportions of effector and central memory T helper 2 cells during acute infection i.e., at disease presentation compared to convalescence, 28 days post-infection defined by a follow-up 28 days after acute infection. However, circulating cytokine-producing (IFN-γ and/or TNF-α) rotavirus-specific VP6-specific CD4^+^ T cells were rarely detectable in children with rotavirus infection at both acute and convalescent stages. Moreover, following whole blood mitogenic stimulation, the responding CD4^+^ T cells were predominantly non-cytokine producers of IFN-γ and/or TNF-α. Our findings demonstrate limited induction of anti-viral IFN-γ and/or TNF-α-producing CD4^+^ T cells in rotavirus-vaccinated Malawian children following the development of laboratory-confirmed rotavirus infection.

## Introduction

Group A rotavirus infection remains the leading cause of severe diarrhoea amongst infants and young children worldwide^1^. It results in approximately 128,500 deaths globally, with over 70% of these cases occurring in sub-Saharan Africa^2^. Rotavirus vaccines have significantly reduced the number of rotavirus-associated deaths; however, vaccination is less effective in low to middle-income countries (LMICs), where the disease burden is greatest, compared to high-income countries^3^. In Malawi, the effectiveness of the Rotarix^®^ vaccine against severe rotavirus diarrhoea following its nationwide introduction in 2012 is approximately 64%^4,5^ which has been associated with a decline in diarrhoea-associated infant mortality by a third^6^ and the reduction in the number of diarrhoea-related hospitalizations by 43%^4^ amongst infants and young children.

Rotavirus is a non-enveloped double-stranded ribonucleic acid (dsRNA) virus. The genome is enclosed in a triple-layered icosahedral capsid that consists of 11 segments that encode six viral proteins (VP1 to VP4, VP6 and VP7) and six non-structural proteins (NSP1 to NSP5/NSP6)^7^. The inner layer is composed of VP2 which encases VP1, the viral RNA-dependent RNA polymerase and VP3, the viral capping enzyme. The middle layer is composed of VP6 which is highly immunogenetic and determines the rotavirus groups^8,9^. VP4 is protease sensitive while VP7 is the glycoprotein comprising the outer layer and is capable of eliciting neutralizing antibodies independently ^10,11^. NSP1 to NSP3, NSP5 and NSP6 are involved in rotavirus replication. NSP4 is involved in morphogenesis and is also called an enterotoxin that mediates some early rotavirus disease events, and it may be the protein that induces antibodies during infection^10,12^. Rotavirus structural and non-structural proteins activate the intestinal dendritic cells during the infection. Activated dendritic cells can stimulate rotavirus-specific T cells to produce cytokines, presumably through cytokine production^13,14^.

The first rotavirus infection in children is most likely to result in severe disease, with subsequent infections associated with a progressively decreasing risk of severe disease outcomes^15^. Symptomatic and asymptomatic infections confer similar degrees of protection^16^. Primary rotavirus infection induces the production of mainly serotype-specific antibodies, but reinfections create a broader immune response including the production of cross-reactive heterotypic antibodies and rotavirus-specific CD4^+^ T cells^17–19^. Anti-rotavirus immunoglobulin A (RV-IgA) against rotavirus antigen viral proteins (VP7 or VP4) are thought to be the primary correlate of protection^20–25^. It is thought that intestinal RV-IgA is necessary for luminal control of rotavirus infection^23^. However, it has been shown that mice can recover from rotavirus infection even in the absence of a strong antibody response, demonstrating the importance of cell-mediated immunity^26^. Rotavirus-specific CD4^+^ T cells are thought to be vital for the development of a protective immune response against rotavirus infection^24^. Previous studies have demonstrated detectable levels of circulating rotavirus-specific T helper cells after rotavirus infection in children^27^. In mouse models, memory VP6-specific CD4^+^ T cells confer protection without the induction of neutralizing antibodies^23^. Together, these observations highlight the potentially important contribution of cell-mediated immunity in defence against rotavirus infection.

Rotavirus-specific T cells play a role in the clearance of rotavirus infection, and these have been detected in both children and adults during the convalescent phase of rotavirus infection^28,29^. Poor induction of CD4^+^ T cells during rotavirus infection leads to limited antibody production which results in deficient elimination of the rotavirus infection^28^. CD4^+^ T cell-derived cytokines stimulate B cells and promote B cell proliferation and immunoglobulin class switching^30^. B cells are a critical component of protective immunity to rotavirus. B cell immunodeficient mice were unable to generate long-term protection against rotavirus^23,31^. Stimulated rotavirus-specific CD4^+^ and CD8^+^ T cells from healthy adults were unable to provide long-term immunity following rotavirus infection because they were terminally differentiated but little is known about CD4^+^ and CD8^+^ T cells in under-five children^32^.

Rotavirus vaccines are the most effective public health strategy in preventing rotavirus gastroenteritis and reducing the rotavirus disease burden^17,19^. Here, we sought to characterize acute and convalescent CD4^+^ T cell responses in Rotarix^®^-vaccinated Malawian children who were hospitalized with rotavirus-positive and rotavirus-negative diarrhoea. We also described the in vitro mediated cytokine-producing CD4^+^ T cell in response to viral protein 6 (VP6) recombinant protein stimulation. We hypothesized that rotavirus infection induces protection via the production of cytokines that favours the inflammatory milieu thereby enabling activation of pre-existing rotavirus vaccine-induced effector and memory T cells. These findings provide insight into our understanding of the circulating rotavirus-specific CD4^+^ T cells during rotavirus infection that could be targeted to enhance vaccine responses.

## Results

### Demographic and clinical characteristics

We recruited a total of 35 children presenting with severe rotavirus-positive (*n* = *19*) and rotavirus-negative diarrhoea (*n* = *16*) (Table 1) at the Queen Elizabeth Central Hospital (QECH), Blantyre, Malawi. The median age (12 vs 12 months, *p* = 0.62) and sex distribution (Female 56% vs 53%, *p* = 0.85) between the respective study groups were similar. All children, except one child with rotavirus-negative diarrhoea who missed the second dose of the vaccine, received both doses of the Rotarix^®^ rotavirus vaccine at weeks 6 and 10 of age as part of the Expanded Programme on Immunization (EPI) schedule. Duration of vomiting was shorter amongst children presenting with rotavirus-positive diarrhoea compared to rotavirus-negative diarrhoea (3 vs 2 days, *p* = 0.028).

**Table 1:** Demographics and clinical characteristics of the study participants.

Characteristic	RV− diarrhoea, N = 16^1^	RV + diarrhoea, N = 19^1^	p-value^2^
Age (months)	12 (0.0, 24.0)	12 (0.0, 36.0)	0.62
Birth weight	3.0 (2.0, 4.0)	3.0 (2.0, 4.0)	0.85
Gender			> 0.99
Female	9/16 (56%)	10/19 (53%)	
Male	7/16 (44%)	9/19 (47%)	
Mother HIV status			0.70
HIV uninfected	12/16 (75%)	12/19 (63%)	
HIV infected	4/16 (25%)	7/19 (37%)	
Weight (kgs)	9.6 (5.6, 11.6)	8.1 (5.4, 11.9)	0.41
Height (cm)	73.8 (62.3, 89.3)	70.0 (58.3, 87.3)	0.10
MUAC (cm)	13.3 (10.2, 15.0)	13.5 (10.2, 15.3)	0.53
Duration of vomiting	3.0 (2.0, 7.0)	2.0 (1.0, 3.0)	0.028
Unknown	6	2	
Duration of diarrhoea	3.0 (2.0, 7.0)	3.0 (2.0, 5.0)	0.024
Duration of fever	0.0 (0.0, 1.0)	1.0 (0.0, 1.0)	0.11

*RV* Rotavirus.

^1^Median (Range); n/N (%).

^2^Wilcoxon rank sum test; Pearson's Chi-squared test.

### Enteric pathogen prevalence in stool samples

Stool samples collected from 54% (19/35) of the study participants at the time when the children presented with disease at QECH tested positive for rotavirus on Enzyme Immunoassay (EIA). We used the real-time PCR analysis platform, the enteric TaqMan Array Cards (TAC) to screen for the presence of other enteric pathogens in the stool samples. Depending on the availability of the samples, TAC confirmed the presence of rotavirus in all children (16/16) with EIA-confirmed rotavirus diarrhoea. Several coinfections were observed among the study participants and there were no differences in the detection rates of coinfecting enteric pathogens between children with rotavirus-negative and positive diarrhoea except for Enteropathogenic *Escherichia coli*_eae (p = 0.0184) and GII Noroviruses (*p* = 0.0445) of which the detection rates were higher in the former (Table 2).

**Table 2:** TaqMan Array Cards PCR results for the children with rotavirus negative and positive diarrhoea.

Pathogen	Rotavirus− No. % (n = 17)	Rotavirus+ No. % (n = 16)	p value
Adenovirus	1 (5.9)	0 (0)	> 0.9999
Aeromonas	0 (0)	1 (6.3)	0.4848
Ancylostoma	0 (0)	0 (0)	NA
Ascaris	0 (0)	0 (0)	NA
Astrovirus	0 (0)	0 (0)	NA
Cclostridium difficile	0 (0)	0 (0)	NA
Campylobacter_16S	4 (23.5)	1 (6.3)	0.2129
Campylobacter_23S2075A	6 (35.3)	3 (18.8)	0.3387
Campylobacter_coli	1 (5.9)	1 (6.3)	> 0.9999
Campylobacter_jejuni	2 (11.8)	1 (6.3)	> 0.9999
Campylobacter_jejuni_coli	3 (17.6)	1 (6.3)	0.5347
Campylobacter_pan	5 (29.4)	3 (18.8)	0.4486
Cryptosporidium	3 (17.6)	0 (0)	0.2273
Cyclospora	0 (0)	0 (0)	NA
*Enterocytozoon bieneusi*	0 (0)	1 (6.3)	0.4848
E_histolytica	0 (0)	0 (0)	NA
*Entamoeba histolytica*	1 (5.9)	0 (0)	> 0.9999
*Encephalitozoon intestinalis*	0 (0)	0 (0)	NA
Enteroaggregative *Escherichia coli* _aaiC	4 (23.5)	1 (6.3)	0.1548
Enteroaggregative *Escherichia coli* _aatA	5 (29.4)	5 (31.3)	0.9791
Enteropathogenic *Escherichia coli_*bfpA	4 (23.5)	1 (6.3)	0.3353
Enteropathogenic *Escherichia coli* _eae	6 (35.3)	0 (0)	0.0184
Enterotoxin-producing *Escherichia coli*_CFA_I	1 (5.9)	1 (6.3)	0.7424
Enterotoxin-producing *Escherichia coli* _CS1	0 (0)	0 (0)	NA
Enterotoxin-producing *Escherichia coli* _CS2	0 (0)	0 (0)	NA
Enterotoxin-producing *Escherichia coli* _CS3	0 (0)	0 (0)	NA
Enterotoxin-producing *Escherichia coli* _CS5	0 (0)	0 (0)	NA
Enterotoxin-producing *Escherichia coli* _CS6	0 (0)	0 (0)	NA
Enterotoxin-producing *Escherichia coli* _LT	2 (11.8)	4 (25.0)	0.3433
Enterotoxin-producing *Escherichia coli* _STh	1 (5.9)	0 (0)	0.4848
Enterotoxin-producing *Escherichia coli* _STp	0 (0)	0 (0)	NA
Giardia	0 (0)	0 (0)	NA
Helicobacter pylori	0 (0)	2 (12.5)	0.2273
Isospora	0 (0)	0 (0)	NA
Mycobacterium tuberculosis	0 (0)	0 (0)	NA
Necator	0 (0)	0 (0)	NA
Norovirus_GI	0 (0)	0 (0)	NA
Norovirus_GII	5 (29.4)	0 (0)	0.0445
Plesiomonas	0 (0)	1 (6.3)	0.4848
Rotavirus	0 (0)	16 (100)	< 0.0001
Salmonella	2 (11.7)	0 (0)	0.4848
Sapovirus-I-II-IV	0 (0)	1 (6.3)	0.4848
Sapovirus-V	0 (0)	0 (0)	NA
Shigella_clade_1	0 (0)	2 (12.5)	0.2273
Shigella_enteroinvasive *Escherichia coli*	3 (17.6)	1 (6.3)	0.3436
Shigella_flexneri_6	0 (0)	1 (6.3)	0.4848
Shigella_flexneri_non6	0 (0)	0 (0)	NA
Shigella_sonnei	0 (0)	1 (6.3)	0.4848
Shiga toxin producing *Escherichia Coli*_stx1	1 (5.9)	1 (6.3)	> 0.9999
Shiga toxin producing *Escherichia Coli* _stx2	0 (0)	0 (0)	NA
Strongyloides	0 (0)	0 (0)	NA
Trichuris	0 (0)	0 (0)	NA
*Vibro cholerae*	0 (0)	0 (0)	NA

### Rotavirus-IgA antibody responses

**Figure 1:**
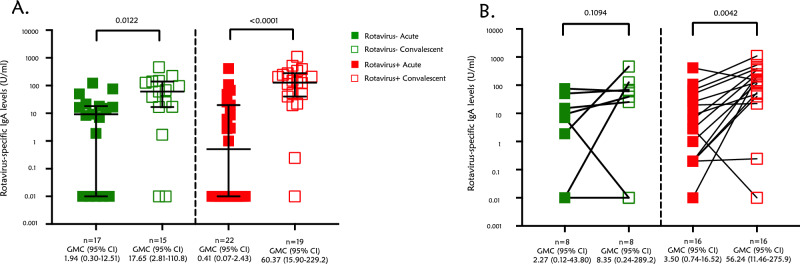
RV-IgA responses in children with rotavirus-positive diarrhoea. RV-IgA was measured from the plasma of the children with laboratory-confirmed rotavirus diarrhoea using a standardized quantitative ELISA. (**A**) RV-IgA geometric mean concentration (U/mL) for children with rotavirus-positive diarrhoea at acute and convalescent phases of infection. (**B**) Paired sample analysis for children with rotavirus-positive diarrhoea at acute and convalescent phases of infection. *CI* confidence interval, *GMC* geometric mean concentration.

We further examined RV-IgA antibody levels in both the acute and convalescent phases sera of the children with rotavirus-negative and rotavirus-positive diarrhoea to determine if they were vaccine failures or were presenting with breakthrough infection. In total, we tested 17 acute and 15 convalescent samples, 22 acute and 19 convalescent samples for IgA obtained from children with rotavirus-negative and rotavirus-positive diarrhoea, respectively. Samples were insufficient in some children, so we had 8 rotavirus-negative and 16 rotavirus-positive diarrhoea paired acute convalescent phase samples. The geometric mean concentration (GMC) levels of IgA was less than 2 U/mL in both Rotavirus-positive and Rotavirus-negative cases at the acute phase which is lower than the seroconversion rate (20 U/mL) suggesting that these were vaccine failures as they were all vaccinated with Rotarix rotavirus vaccine prior to presenting with diarrhoea. Children with rotavirus-positive diarrhoea had 0.41 [0.07–2.43; 95% confidence interval (CI)] GMC of RV-IgA during the acute phase, which was significantly lower compared to their IgA titers at convalescent phase, [GMC of 60.37 (15.90–229.2; 95% CI)], *p* < 0.0001 (Fig. 1A). We observed an increase in rotavirus-specific RV-IgA levels 28 days post-infection (convalescent phase) in almost all children with rotavirus-positive diarrhoea that had paired samples, Fig. 1B. Although the children with rotavirus-negative diarrhoea had detectable RV-IgA antibodies at the convalescent phase of infection, their GMC (95% CI) was not statistically different (*p* = 0.1094) from the levels detected at the acute phase of infection.

### Rotavirus diarrhoea was associated with a higher proportion of the effector and central memory Th2 cells at the acute phase of infection unlike rotavirus-negative diarrhoea

**Figure 2:**
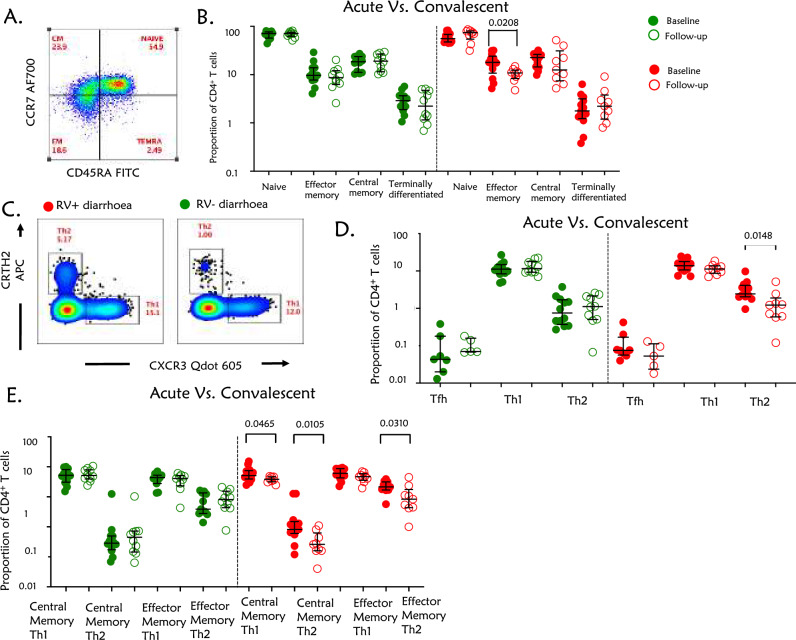
CD4^+^ T subsets in children with or without rotavirus diarrhoea. The cells were stained with the following fluorochrome-conjugated antibodies CD3 PerCP-CY5.5, CD4 APC-CY7, CCR7 AF700, CD45RA FITC, CRTH2 APC, CXCR3 Qdot 605, PD1 PE CY7 and BCL6 PE. Central memory T cells were defined as CD4^+^, CCR7^+^ and CD45RA-, effector memory T cells as CD4^+^, CD45RA^−^ and CCR7^−^, terminally differentiated as CD4^+^, CD45RA^+^ and CCR7^−^ and naïve T cells as CD4^+^, CCR7^+^ and CD45RA^+^. Th1 was defined as CD4^+^ and CXCR3^+^, Th2 as CD4^+^ and CRTH2^+^ and Tfh as CD4^+^ BCL6^+^ and PD1^hi^. Central memory Th1 was defined as CD4^+^, CXCR3^+^, CD45RA^−^ and CCR7^+^, effector memory Th1 as CD4^+^, CXCR3^+^, CD45RA^−^ and CCR7^−^, Central memory Th2 was defined as CD4^+^, CRTH2^+^, CD45RA^−^ and CCR7^+^ and effector memory Th1 as CD4^+^, CRTH2^+^ CD45RA^−^ and CCR7^−^. Mann–Whitney U test was used to compare the proportion of the T cells between children with rotavirus-positive and rotavirus-negative at both acute and convalescent phases of infection. Differences after comparisons were considered statistically significant if p-values were less than 0.05. (**A**) Representative flow cytometry plot from a child with rotavirus diarrhoea peripheral whole blood showing the memory T cell subsets. (**B**) Proportion of naïve, central memory, effector memory and terminally differentiated T cells for children with rotavirus-negative (in Green) diarrhoea at the acute and convalescent phase of infection and children with rotavirus-positive (in Red) diarrhoea at the acute and convalescent phase of infection. (**C**) Representative flow cytometry plot from children with rotavirus-positive diarrhoea and rotavirus-negative diarrhoea peripheral whole blood showing Th1 and Th2 cell subsets. (**D**) Proportion Th1, Th2 and Tfh cells for children with rotavirus-negative (In Green) diarrhoea at the acute and convalescent phase of infection and children with rotavirus-positive diarrhoea (In Red) at the acute and convalescent phase of infection. (**E**) Proportion of central memory Th1, central memory Th2, effector memory Th1 and effector memory Th2 for children with rotavirus-negative (In Green) diarrhoea at the acute and convalescent phase of infection and children with rotavirus-positive (In Red) diarrhoea at the acute and convalescent phase of infection.

We sought to identify the CD4^+^ T cell subsets induced following rotavirus infection in children, (Fig. 2A,C). CD4^+^ T cells develop into populations of effector T cells that migrate to sites of infection^33^. Evidence suggests that effector CD4^+^ T cells have strong protective roles during viral infection that is independent of their helper activities^34^. Using flow cytometry, we measured the proportions of naïve (CD45RA^+^CCR7^+^), effector memory (EM) (CD45RA^−^CCR7^−^), central memory (CM) (CD45RA^−^CCR7^+^), terminally differentiated (TEMRA) (CD45RA^+^CCR7^−^), Th1 (CXCR3^+^), Th2 (CRTH2^+^) and Tfh (BCL6^+^ PD1^hi^) CD4^+^ T cell subsets (Fig. 2B,D). In children with rotavirus-positive diarrhoea, the proportion of effector memory (*p* = 0.0208) and Th2 cells (*p* = 0.0148) was higher during the acute infection compared to the convalescence period, but this was not the case in children presenting with rotavirus-negative diarrhoea (Fig. 2B,D). Furthermore, the proportion of effector memory Th2 (*p* = 0.0310) and central memory Th1 (*p* = 0.0465) and Th2 (*p* = 0.0105) were higher in rotavirus-positive children at the acute stage of infection compared to the convalescence stage, but this was not the case in children with rotavirus-negative diarrhoea (Fig. 2E). These findings indicate that acute rotavirus infection is associated with the induction of circulating memory CD4^+^ T cell subsets, which quickly return to baseline levels following recovery from the disease.

### VP6-specific CD4^+^ T cells are rarely present in children presenting with rotavirus-positive and rotavirus-negative diarrhoea at both acute and convalescent phases of infection

**Figure 3:**
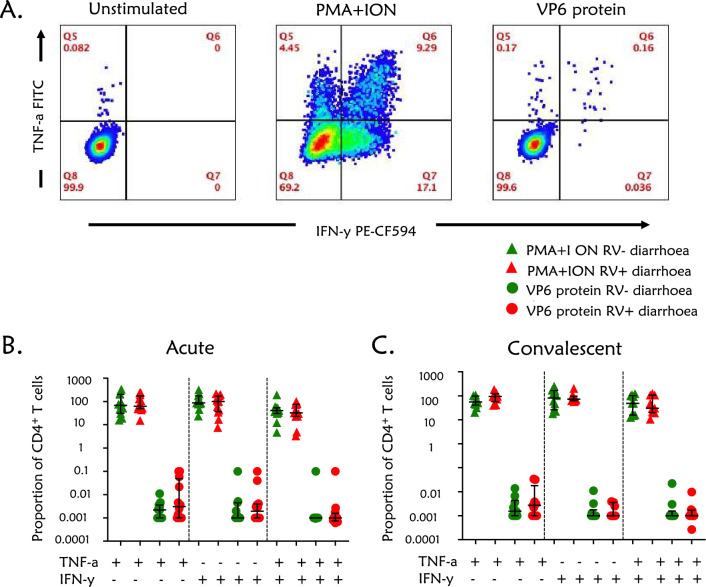
CD4^+^ T stimulations in children with or without rotavirus diarrhoea. The cells were stained with the following fluorochrome-conjugated antibodies CD3 PerCP-CY5.5, CD4 BV 421, CD8 APC-CY7, TNF-α FITC, IFN-γ PE-CF594 and CD69 AF700. Mann–Whitney U test was used to compare the proportion of the T cells between children with rotavirus-positive and rotavirus-negative at both acute and convalescent phases of infection. Differences after comparisons were considered statistically significant if p-values were less than 0.05. (**A**) Representative FACS plot from children with rotavirus diarrhoea peripheral whole blood showing CD4 ^+^ T cells producing TNF-α and IFN-γ cells from PMA + ION (Mitogen) and VP6 protein responses and the unstimulated control. (**B**) Proportion of CD4 ^+^ producing TNF-α or IFN-y and both TNF-α and IFN-γ cells for children with rotavirus rotavirus-positive and rotavirus-negative diarrhoea at the acute phase of infection. (**C**) Proportion of CD4 ^+^ producing TNF-α or IFN-y and both TNF-α and IFN-γ cells for children with rotavirus-positive and rotavirus-negative diarrhoea at the convalescent phase of infection.

We sought to ascertain whether the altered memory CD4^+^ T cell subsets were rotavirus-specific, by assessing the frequency of TNF-α and/or IFN-γ-producing cells at acute and convalescent phases in children with or without rotavirus-confirmed diarrhoea following stimulation with recombinant rotavirus VP6 protein. Representative flow cytometry plots for the unstimulated control, VP6 protein and PMA and Ionomycin (mitogen) gated on CD4^+^ T cells (Fig. 3A). TNF-α and/or IFN-γ-producing VP6-specific CD4^+^ T cells were rarely detected in children presenting with or without rotavirus diarrhoea at both acute and convalescent phases of infection, with frequencies mostly below 0.01% (Fig. 3B,C and Supplementary Fig. 1). Moreover, the frequency of TNF-α and/or IFN-γ-producing VP6-specific CD4^+^ T cells was similar between children presenting with rotavirus-positive diarrhoea compared to rotavirus-negative diarrhoea (Fig. 3B,C). However, TNF-α and/or IFN-γ-producing CD4^+^ T cells were detectable in the mitogen-stimulated conditions (Fig. 3B,C), indicating that the cells were capable of producing TNF-α and/or IFN-γ. Together, these findings indicate that the effector memory CD4^+^ T cells induced by rotavirus infection are unlikely to represent TNF-α or IFN-γ-producing VP6-specific CD4^+^ T cells.

### CD4^+^ T cells are intrinsically monofunctional and biased away from IFN-γ or TNF-α cytokine production in children presenting with or without rotavirus diarrhoea

**Figure 4:**
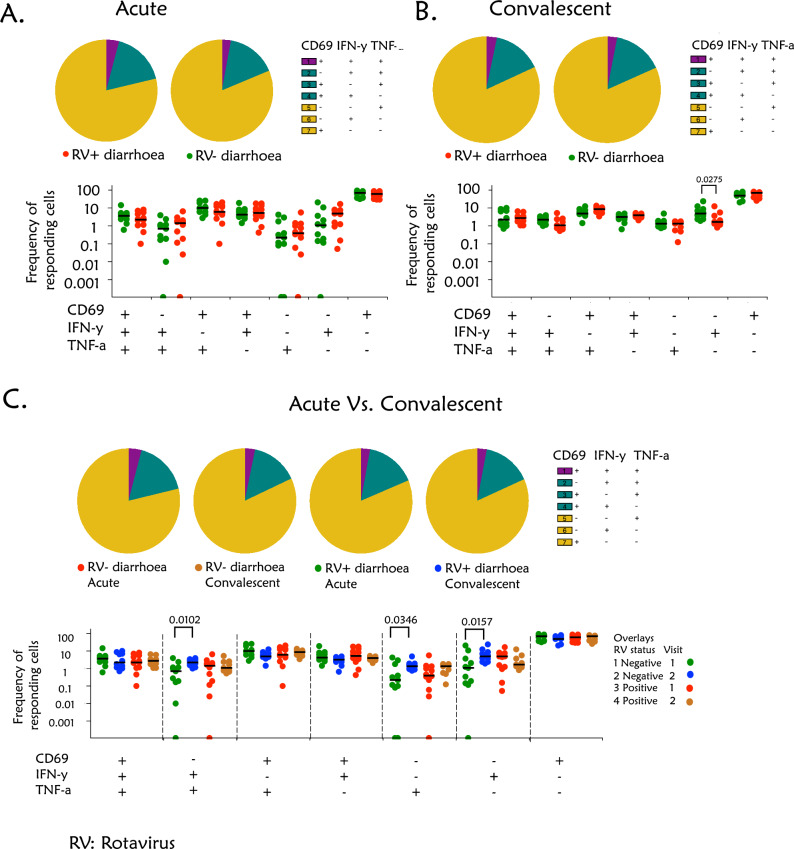
CD4^+^ T cells mitogenic cytokine profiles in children with or without rotavirus diarrhoea. The cells were stimulated with either PMA + ION or VP6 protein for 18 hours, with the unstimulated control in all experiments. The cells were stained with the following fluorochrome-conjugated antibodies CD3 PerCP-CY5.5, CD4 BV421, CD8 APC-CY7, TNF-α FITC, IFN-γ PE-CF594 and CD69 AF700. Single producers were either CD4 ^+^ T cells producing TNF-α or CD4 ^+^ T cells producing IFN-γ and double producers were CD4 ^+^ T cells producing TNF-α and IFN-γ. Mann–Whitney U test was used to compare the proportion of the T cells between children with rotavirus-positive and rotavirus-negative at both acute and convalescent phases of infection. Differences after comparisons were considered statistically significant if p-values were less than 0.05. (**A**) Frequency of CD4 ^+^ T cells producing TNF-α and IFN-γ, activated (CD69^+^) or not activated (CD69^−^) in children with rotavirus-positive and rotavirus-negative diarrhoea at the acute phase of infection. (**B**) Frequency of CD4 ^+^ T cells producing TNF-α and IFN-γ, activated (CD69^+^) or not activated (CD69^−^) in children with rotavirus-positive and rotavirus-negative diarrhoea at the convalescent phase of infection. (**C**) Frequency of CD4 ^+^ T cells producing TNF-α and IFN-γ, activated (CD69^+^) or not activated (CD69^−^) in children with rotavirus-positive and rotavirus-negative diarrhoea at the acute and convalescent phases of infection.

We then sought to further ascertain the intrinsic functional capacity of CD4^+^ T cells from children presenting with or without rotavirus-positive diarrhoea at acute presentation and convalescence. Using Boolean gating of CD69, IFN-γ and TNF-α, we evaluated the functional profile of mitogen-stimulated CD4^+^ T cells. The majority of the responding CD4^+^ T cells were CD69^+^IFN-γ^−^TNF-α^−^ in both rotavirus-positive and rotavirus-negative children with diarrhoea at acute and convalescence phases (Fig. 4A,B). The responding CD4^+^ T cells were rarely polyfunctional (CD69^+^IFN-γ^+^TNF-α^+^) in rotavirus-positive and rotavirus-negative children at acute and convalescence stages (Fig. 4A,B). However, the frequency of CD69^−^IFN-γ^+^TNF-α^−^ CD4^+^ T cells was decreased in the rotavirus-positive children compared to those with rotavirus-negative (Fig. 4B).

Furthermore, the frequency of CD69^−^IFN-γ^+^TNF-α^+^, CD69^−^IFN-γ^−^TNF-α^+^ and CD69^−^IFN-γ^+^TNF-α^−^ CD4^+^ T cells were higher during acute infection compared to the convalescent stage in children with rotavirus-negative diarrhoea (p = 0.0102, p = 0.0157 and p = 0.0346, respectively) (Fig. 4C). In contrast, there were no statistically significant differences in the frequency of mitogen-responsive CD4^+^ T cells between acute and convalescence stages in children with rotavirus-positive diarrhoea (all p > 0.05) (Fig. 4C). Collectively, these data show that the CD4^+^ T cells in these children were predominantly monofunctional and were biased away from TNF-α and/or IFN-γ-production.

## Discussion

Rotavirus-induced CD4^+^ T cells have been shown to mediate anti-viral protection in animal models^34^, but their role in humans is unclear. In this study, we found that acute rotavirus infection was associated with the induction of circulating memory CD4^+^ T cell subsets, which then return to baseline levels during convalescence. However, the rotavirus infection-induced effector memory CD4^+^ T cells were not targeted at the highly immunogenic rotavirus VP6 protein, and they did not produce TNF-α or IFN-γ. Moreover, functional profiling from mitogenic stimulation confirmed that the CD4^+^ T cell responses were biased away from TNF-α and/or IFN-γ-production. This study highlights limited induction of the anti-viral CD4^+^ T cell responses in Malawian children.

The scarcity of circulating rotavirus VP6-specific CD4^+^ T cells producing IFN-γ and TNF-α in rotavirus-vaccinated children following rotavirus infection was unexpected. However, previous studies have also found low frequencies of rotavirus-specific T cells producing IFN-γ in children with rotavirus infection^23,35^. It has also been observed that the frequencies of circulating rotavirus-specific CD4^+^ T cells producing IFN-γ, TNF-α, or IL-2 in children are lower than those producing cytokines specific to tetanus toxoid and influenza virus^35^. The observed low frequency of IFN-γ and TNF-α producing circulating rotavirus-specific CD4^+^ T cells could be due to their sequestration in the intestine or other organs, away from the systemic circulation, as suggested in the murine model^36^. Alternatively, this could be due to differential polarization mediated by a distinct cytokine microenvironment in children. CD4^+^ T cell stimulation in the presence of transforming growth factor β (TGF-β) favours Th2 differentiation and proliferation of CD4^+^ T cells that do not display immediate cytokine production capacity^37,38^. Therefore, it is plausible that rotavirus-specific CD4^+^ T cells primed at the site of infection, the gastrointestinal tract, have a unique cytokine production profile or differential trafficking potential, leading to their rarity in circulation.

CD4^+^ T cell cytokine production is an essential component of the helper function, and the ability to produce multiple cytokines, termed polyfunctionality, has been associated with protective immunity^39^. Polyfunctional CD4^+^ T cells have been shown to be beneficial in immune responses against *Leishmania major*^40^, HIV^41^, HCV^36^, dengue virus^35^, and *Mycobacterium tuberculosis*^42^. In our study, we have observed poor polyfunctionality of CD4^+^ T cells in general, let alone in the rotavirus VP6-specific CD4^+^ T cells. How this poor polyfunctional capacity of CD4^+^ T cells impacts susceptibility to severe rotavirus diarrhoea is unknown and warrants further investigation. Furthermore, it remains unclear how well circulating rotavirus VP6-specific CD4^+^ T cell responses represent at the site of infection and how they are directly involved in anti-viral clearance.

RV-IgA levels have been shown to increase with repeated natural rotavirus infection and were associated with reduced risk of future rotavirus gastroenteritis^43^. We detected low levels (< 20 U/ml) of RV-IgA at the acute phase of infection in children with or without rotavirus-associated diarrhoea. RV-IgA levels increased significantly in convalescent sera in almost all children presenting with rotavirus-positive diarrhoea unlike in those that presented with rotavirus-negative diarrhoea. Lower RV-IgA measured during the acute phase of infection was strongly associated with increased odds of Rotarix® clinical vaccination failure in Malawi^44^. Our current findings offer additional proof that RV-IgA is linked to clinical protection at both the individual and population levels^44^. These findings demonstrated that natural infection induces significant levels of RV-IgA post-infection, thus, justifying why IgA is used as a proxy marker of induced immune protection against rotavirus exposure as the true correlate of protection is not known. Although the GMC IgA levels were lower than the seroconversion rate (20 U/mL) in rotavirus-negative cases at the convalescence phase, we observed an increment in IgA titres in at least 2 participants. This could potentially be due to nosocomial infection as both rotavirus-positive and negative cases were hospitalized in the same pediatrics ward at QECH or they acquired the infection in the community post-hospital discharge as the force of rotavirus infection is quite high in this setting^45^.

The study had several limitations. Firstly, it is plausible that VP6 protein is not the optimal antigen for detecting vaccine or infection rotavirus-specific CD4^+^ T cells in humans, despite it being the most abundant and immunogenic rotavirus protein^46^. The use of multiple rotavirus antigens for stimulation or the use of specific tetramers that do not depend on the cytokine-secreting ability of antigen-specific cells could have potentially increased our probability to detect other rotavirus-specific CD4^+^ T cells of different functional profiles and specificities. Secondly, this study focused on very sick children with rotavirus diarrhoea, who since they had received the rotavirus vaccine could be classified as rotavirus vaccine failures, hence it is possible that our results are biased towards a group of children that have impaired rotavirus-specific CD4^+^ T cell responses. However, the fact that rotavirus-specific CD4^+^ T cells were also rarely detected in rotavirus-vaccinated children presenting with rotavirus-negative diarrhoea suggests that this is unlikely to have been the major reason.

In conclusion, our study shows that natural rotavirus infection induced RV-IgA responses in rotavirus-vaccine failures and there is an association between rotavirus infection and heightened T cell immune responses. Furthermore, we have demonstrated that circulating rotavirus VP6-specific CD4^+^ T cells are rarely detected in rotavirus-vaccinated Malawian children with severe rotavirus diarrhoea. The findings may suggest that poor induction of IFN-γ and TNF-α producing rotavirus VP6-specific CD4^+^ T cells could contribute to rotavirus vaccine failure and could in part explain the reduced effectiveness of rotavirus vaccines in children from low-income, high-disease-burdened settings.

## Materials and methods

### Study recruitment

We recruited children ≤ 5 years of age presenting at the Queen Elizabeth Central Hospital (QECH) with severe rotavirus-positive and rotavirus-negative diarrhoea. Following informed consent, peripheral blood and stool samples were collected from children who fulfilled the inclusion and exclusion criteria at the time of disease presentation (acute phase) and 28 days later post-infection (convalescent phase). The inclusion criteria included: children ≤ 5 years of age; children’s clinical illness was not explained by an alternative underlying condition; children’s clinical illness commenced within 14 days before the hospital visit; children were screened for rotavirus, and children were seen and or admitted at QECH and lived in Blantyre district. Exclusion criteria included: children ≥ 5 years of age; children that we were unable to contact their parents or guardian to obtain informed consent; children admitted to another hospital for over 24 h and subsequently transferred to QECH; children who presented within 14 days of the previous hospital for the same illness; children hospitalized > 48 h before enrollment, children with an oncological diagnosis or congenital immunodeficiency (apart from HIV) and lived outside Blantyre district. The study was approved by the College of Medicine Research Ethics Committee, Malawi (COMREC, reference number P.10/20/3154) and the National Health Sciences Research Committee, Malawi (NHSRC, protocol number #867).

### Diagnosis and confirmation of rotavirus infection

Stool samples were initially screened for the presence of rotavirus using a rotavirus-specific Rapid Diagnosis Test (RDT) (InTec, Xiamen, China). This helped to stratify children into rotavirus-positive and rotavirus-negative groups during recruitment. The rotavirus diagnosis was confirmed using a Premier® Rotaclone® Enzyme Immunoassay (EIA) (Meridian Biosciences, Cincinnati, OH, USA) and enteric Taqman Array Cards (TAC) real-time polymerase chain reaction (PCR) assay^47^.

### Rotavirus-specific IgA responses measurement

RV-IgA antibodies were detected from the serum samples using standardized quantitative enzyme-linked immunosorbent (ELISA) assay^48–50^. These methods have been validated and re-certified for regulatory submissions for rotavirus vaccine trials.

### Screening of various enteric pathogens associated with diarrhoea

TaqMan Array Card to detect 42 enteropathogens, including viruses (adenovirus, astrovirus, norovirus, rotavirus, and sapovirus), bacteria (Campylobacter jejuni/C. coli, Clostridium difficile, Salmonella, Vibrio cholera, diarrheagenic Escherichia coli strains including enteroaggregative *E. coli* [EAEC], enterotoxigenic *E. coli* [ETEC], enteropathogenic *E. coli* [EPEC], and Shiga-toxigenic *E. coli* [STEC]), Shigella/enteroinvasive *E. coli* (EIEC), Shigella flexneri/sonnei, mycobacterium tuberculosis, Plesiomonas, Helicobacter pylori and Aeromonas), protozoa (Cryptosporidium, *Encephalitozoon intestinalis,* Cyclospora, Entamoeba histolytica, *Enterocytozoon bieneusi*, Isospora and Entamoeba histolytica), and helminths (Ascaris, Necator, Ancylostoma, Strongyloides and Trichuris), as well as two extrinsic controls to monitor extraction and amplification efficiency. The TAC method performance has been previously described; samples were classified as pathogen positive at a cycle threshold of < 35^51,52^.

### Flow cytometry assays

#### Immunophenotyping

Whole blood samples were stained with antibodies to CD4^+^ T cell phenotypes based on the markers on the surface of the cells. The following antibodies were used for the staining panel, CD3 PerCP-CY5.5 (HIT3a, BioLegend, United Kingdom (UK)), CD4 APC-CY7 (RPA-T4, BD Pharmingen, UK), CCR7 AF700 (G043H7, BioLegend, UK), CXCR3 BV605 (G025H7, BioLegend, UK), CRTH2 APC (BM16, BioLegend, UK), CXCR5 BV421(J252D4, BioLegend, UK), PD1 PE CY7 (EH12.2H7, BioLegend, UK), CD45RA FITC (H1100, BD Pharmingen, UK) and BCL6 PE (IG191E/AS, BioLegend, UK). After staining, the cells were lysed with diluted 10X BD FACS lysing solution (BD Biosciences, UK) before acquisition on the flow cytometry.

CD4^+^ T cells were identified as CD3^+^, and CD4^+^ and all subsets below were derived from that defined population. Central memory T cells were defined as CD4^+^, CCR7^+^ and CD45RA^−^, effector memory T cells as CD4^+^, CD45RA^−^ and CCR7^−^, terminally differentiated as CD4^+^, CD45RA^+^ and CCR7^−^ and naïve T cells as CD4^+^, CCR7^+^ and CD45RA^+^. Th1 was defined as CD4^+^ and CXCR3^+^, Th2 as CD4^+^ and CRTH2^+^ and Tfh as CD4^+^ BCL6^+^ and PD1^hi^. Central memory Th1 was defined as CD4^+^, CXCR3^+^, CD45RA^−^ and CCR7^+^, effector memory Th1 as CD4^+^, CXCR3^+^, CD45RA^−^ and CCR7^−^, central memory Th2 was defined as CD4^+^, CRTH2^+^, CD45RA^−^ and CCR7^+^ and effector memory Th1 as CD4^+^, CRTH2^+^ CD45RA^−^ and CCR7^−^ (Supplementary Fig. 2).

#### Stimulation assay

Whole blood (100 ul) was stimulated with Viral protein 6 (VP6), 0.1 mg in tris budder with slats of pH 7.5 or cell activation cocktail (423301, BioLegend, UK) composed of Phorbol 12-myristate 13-acetate (PMA) 40.5 µM and Ionomycin (ION) combination 669.3 µM, with an unstimulated sample as a negative control. PMA and ION combination is a good stimulant for cytokine-stimulating cells and was used as the positive control^32^. VP6 protein (DAGA-3002) was supplied by CD creative diagnostics, USA. VP6 protein is a recombinant protein, highly immunogenic derived from *Escherichia Coli* used as an immunogen for rotavirus-specific responses. This VP6 was derived from rotavirus A, strain RVA/Human/United Kingdom/ST3/1975/G4P2A[6]. The stimulation volumes for the cell activation cocktail comprising PMA+ION and VP6 were 1 ul and 0.6 ul respectively and these were titrated and optimized on volunteers' human blood samples in the laboratory before use. These stimulation volumes were determined following titration and optimization. All responses were background subtracted from an unstimulated control.

The simulations were done in 96-well culture plates and incubated for 18 h at 37 °C with 5% carbon dioxide (C0_2_). The last 12 h of the incubation included brefeldin A (BD Biosciences, UK) and monensin (BD Biosciences, UK) to block the secretion of cytokines from the cells. At the end of the incubation, the cells were harvested and stained with CD3 PerCP-CY5.5 (HIT3a, BioLegend, UK), CD8 APC-CY7 (HIT8a, BioLegend, UK), CD4 BV421 (RPA-T4, BioLegend, UK) and CD69 AF700 (FN50, BD Pharmingen, UK) to identify CD69^−^IFN-γ^+^TNF-α^+^, CD69^−^IFN-γ^−^TNF-α^+^ and CD69^−^IFN-γ^+^TNF-α^−^ CD4^+^ T cells. Red blood cells (RBC) were lysed with 3 ml of 10X BD FACS lysing solution supplied by BD Biosciences, UK. Cells were then permeabilized and washed using a transcription factor buffer set (Thermofisher Scientific, UK) and stained with TNF-α FITC (Mab 11, BioLegend, UK) and IFN-γ PE-CF594 (B27, BD Biosciences, UK). Lastly, the cells were acquired (at least 100,000 events in the CD4^+^ T cell gate) on an LSRFortessa flow cytometer equipped with FACSDIVA software (BD Biosciences, UK). All flow cytometry data were analyzed using FlowJo software (version 10.8.1, BD Biosciences, UK) (See Supplementary Fig. 3, gating strategy).

### Laboratory experiments

All experiments were performed at Malawi Liverpool Wellcome Research Programme (MLW) laboratory in accordance with relevant guidelines and regulations. MLW laboratory is ISO 15180:2012 accredited by the Southern African Development Community Accreditation Service (SADCAS).

### Statistical analysis

Statistical analyses and graphical presentations were done using GraphPad Prism 9 (GraphPad Software, San Diego California, USA), Pestle version 2.0 (20019, Vaccine Research Center, NIAID, USA), Spice 6 version 6.1 (61001, Vaccine Research Center, USA) and R version 4.1.3 (Free software Foundation’s GNU General Public License. Mann–Whitney U test was used for non-paired data and results were given as median with range and number with a percentage. Differences after comparisons were considered statistically significant if p-values were less than 0.05.

### Confirmation of informed consent

The studies ensured that the consent process was undertaken in an appropriately private environment and questions from parents and or legal guardians were encouraged and answered properly by research nurses. Informed consent for parents and/or legal guardians that joined the study was documented by signature and an inked thumbprint for the illiterate in the presence of the impartial witness. Finally, the consent form was signed by the research nurse obtaining consent, to confirm the study has been fully explained. A copy of the study information sheet and signed informed consent form were offered to the parents and or legal guardians.

## Supplementary Information

Supplementary Figures.

## Data Availability

The datasets analysed in this study are available from the corresponding author on reasonable request.
