# Supplementary material for: Acute rotavirus infection is associated with the induction of circulating memory CD4+ T cell subsets
Source: Sci Rep. 2023 Jun 2;13:9001. doi: 10.1038/s41598-023-35681-9 (PMC10238530; doi:10.1038/s41598-023-35681-9)
Supplement: Supplementary file 1 — Supplementary Figures. [file 41598_2023_35681_MOESM1_ESM.docx]

**Supplementary material**

**Supplementary Figure 1: CD4^+^ T cells stimulations for VP6 responders.**  Peripheral whole blood samples from children with rotavirus-positive and rotavirus-negative diarrhoea for the VP6 responders following ex vivo stimulations. The cells were stimulated with PMA+ION and VP6 protein for 18 hours, with the unstimulated control in all experiments. The cells were stained with the following fluorochrome-conjugated antibodies CD3 PerCP-CY5.5, CD4 BV421, CD8 APC-CY7, TNF-α FITC, IFN-γ PE-CF594 and CD69 AF700. Single producers were either CD4^+^ T cells producing TNF-α or CD4^+^ T cells producing IFN-γ and double producers were CD4^+^ T cells producing TNF-α and IFN-γ.


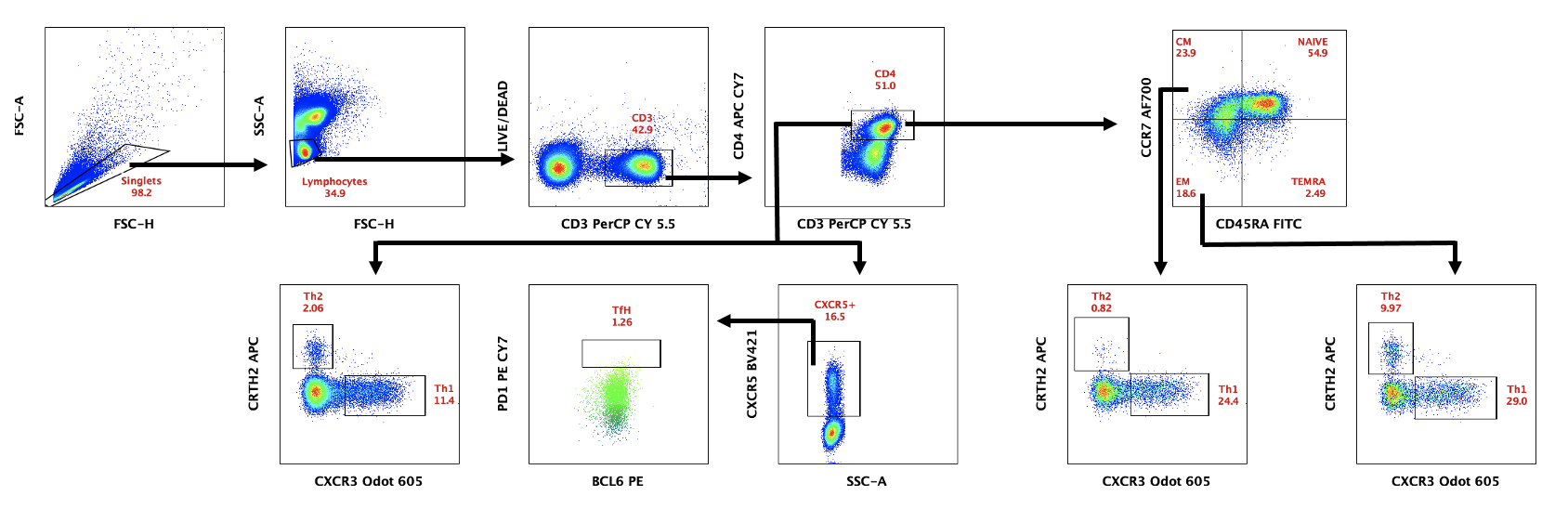


**Supplementary Figure 2: CD4^+^ T cell subsets and memory phenotype gating strategy.** A representative sample from a child with rotavirus diarrhoea peripheral whole blood. The cells were stained with the following fluorochrome-conjugated antibodies CD3 PerCP-CY5.5, CD4 APC-CY7, CCR7 AF700, CD45RA FITC, CRTH2 APC, CXCR3 Qdot 605, PD1 PE-CY7 and BCL6 PE. Central memory (CM) T cells were defined as CD4^+^ T cells expressing CCR7, effector memory (EM) T cells as CD4^+^ T cells not expressing CD45RA and CCR7, terminally differentiated (TEMRA) as CD4^+^ T cells expressing CD45RA and naïve T cells as CD4^+^ T cells expressing CCR7 and CD45RA. Th1 was defined as CD4^+^ cells expressing CXCR3, Th2 as CD4^+^ T cells expressing CRTH2 and Tfh as CD4^+^ T cells expressing BCL6 and PD1^hi^.

**
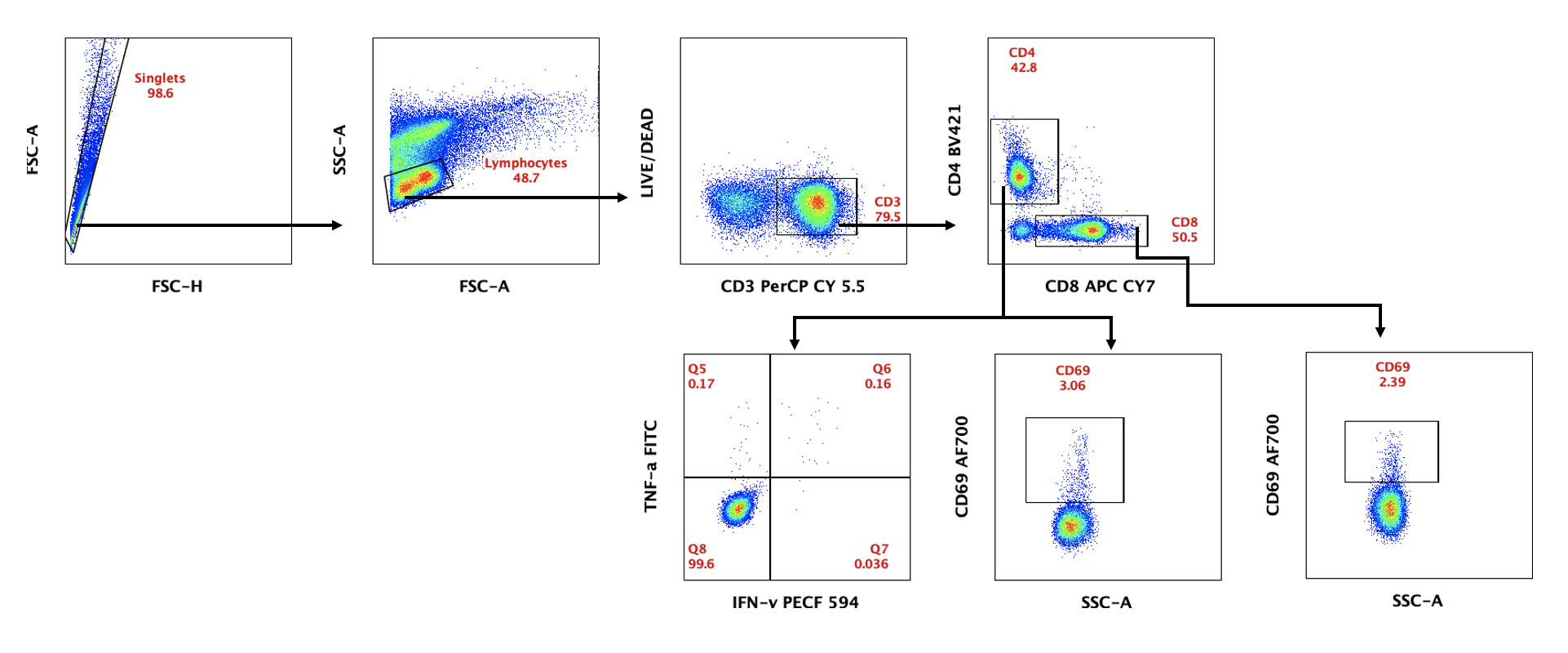
**

**Supplementary Figure 3: CD4^+^ T cells stimulations gating strategy.** A representative sample from a child with rotavirus diarrhoea peripheral whole blood. The cells were stained with the following fluorochrome-conjugated antibodies CD3 PerCP-CY5.5, CD4 BV 421, CD8 APC-CY7, TNF-α FITC, IFN-γ PE-CF594 and CD69 AF700. Single producers were either CD4^+^ T cells producing TNF-α or CD4^+^ T cells producing IFN-γ; double producers were CD4^+^. T cells produce TNF-α and IFN-γ. We further defined activated CD4^+^ T cells as CD4^+^ CD69^+^ and CD8^+^ CD69^+^.
